# Helpful elements in a brief work-oriented intervention targeting musculoskeletal and mental health literacy

**DOI:** 10.1093/heapro/daad132

**Published:** 2023-10-21

**Authors:** Tone Langjordet Johnsen, Torill Helene Tveito, Irene Øyeflaten

**Affiliations:** Division of Physical Medicine and Rehabilitation, Vestfold Hospital Trust, POB 2168, 3103 Tønsberg, Norway; Department of Health, Social and Welfare Studies, University of South-Eastern Norway, Raveien 215, 3184 Horten, Norway; Department of Health, Social and Welfare Studies, University of South-Eastern Norway, Raveien 215, 3184 Horten, Norway; NORCE Norwegian Research Centre, Nygårdsgaten 112, 5008 Bergen, Norway

**Keywords:** health literacy, health promotion, health education, health communication, subjective health complaints

## Abstract

Musculoskeletal and mental health complaints are common in the general population and frequent reasons for healthcare utilization and work absence. Illness perceptions, coping expectancies, rumination and self-stigma are important factors in the management of these health complaints and factors closely linked to health literacy (HL). The aims of the study were to identify helpful elements in a brief intervention (BI) targeting HL regarding common musculoskeletal and mental health complaints and to identify patient perceptions of how the intervention was helpful and whether it affected their subsequent coping. Three focus group interviews with 14 patients were conducted. Systematic text condensation was used for the analysis, supported by the health literacy skill (HLS) framework to sharpen the focus on intervention elements related to the acquisition and utilization of HLSs. Results revealed the importance of receiving comprehensible health information and guidance, the use of metaphors to create recognizable narratives and the use of practical examples and exercises. Normalizing the experienced health complaints, together with a safe and accommodating clinical environment, facilitated the change process. The BI initiated processes that contributed to acceptance, resilience and empowerment, aiding work–life balance and return to work. The study presents authentic narratives of value for future focus in BI.

**Contribution to Health Promotion**

- Highlights the importance of focusing on patients’ health literacy skills in healthcare interventions
- Identifies intervention elements that may promote the development, enhancement and application of health literacy skills among patients
- Highlights the importance of healthcare provider communication skills in health education interventions

## INTRODUCTION

Musculoskeletal and mental disorders are prevalent in the general population (Kessler *et al.*, 2005; Sebbag *et al.*, 2019) with a high degree of comorbidity (Reme *et al.*, 2011). In Norway, more than 50% of employees who are sick-listed from their work have a diagnosis of musculoskeletal or mental disorders (NAV, 2022). A large proportion of these disorders may be classified as subjective health complaints or medically unexplained symptoms (Waddell and Burton, 2005; Ursin and Eriksen, 2006). Reasons for work absences and reduced function among these employees are multifactorial, and may include biological, psychological and social factors that interact and mutually influence each other (Waddell and Burton, 2005; Ursin and Eriksen, 2006). In the management of these health complaints, illness perceptions (Petrie and Weinman, 2006; Hoving *et al.*, 2010), coping expectancies (Ree *et al.*, 2014), rumination (Verkuil *et al.*, 2007) and self-stigma (Schnyder *et al.*, 2017) are addressed as important targets for intervention. Patients’ beliefs and expectancies are vital elements in all these factors (Ursin and Eriksen, 2004; Petrie and Weinman, 2006; Verkuil *et al.*, 2007; Schnyder *et al.*, 2017), and these factors may be influenced through health education, an essential component in influencing people’s health literacy (HL) (Nutbeam, 2000). Psychoeducational interventions seem promising to reduce symptoms of depression and psychological distress for individuals with psychological disorders (Donker *et al.*, 2009). Similarly, education targeting pain neuroscience is effective in reducing pain and disability for individuals experiencing low back pain (Ma *et al.*, 2023). The effort needed to improve people’s HL is receiving growing attention due to its significant benefits to individual and public health and to improve the sustainability of healthcare systems (Nutbeam, 2000; Institute of Medicine Committee on Health Literacy, 2004; Sørensen *et al.*, 2012, 2021; WHO, 2021).

HL is considered a key determinant of health (Kickbusch *et al.*, 2013) and a central target area in the field of health promotion (WHO, 2013). HL involves both personal learning and skills, including the ability to navigate complex social and health systems and the ability to support others in healthy actions (Nutbeam, 2008; WHO, 2013). HL is closely linked to empowerment, a distinct but intertwined concept, and both concepts are central in the health behavior literature (Schulz and Nakamoto, 2013). Systematic reviews confirm the association between HL and positive health behavior (DeWalt and Hink, 2009; Stormacq *et al.*, 2020; Walters *et al.*, 2020). Having a higher degree of mental HL, for example, is associated with less stigmatizing and more positive attitudes toward people with mental illness, more positive attitudes to seeking help for mental health problems and more positive coping behavior (Svensson and Hansson, 2016; Jung *et al.*, 2017; Song *et al.*, 2023). Among musculoskeletal and chronic pain patients, higher HL is associated with less pain intensity, lower fear of movement and better disease-related knowledge and beliefs about pain (Köppen *et al.*, 2018; Mackey *et al.*, 2019; Bittencourt *et al.*, 2021). Little research has examined the connection between HL and work participation but the study of Gernert *et al.* (2022) found an association between HL and work ability (HL explained 17.5% of the variance in the participants’ work ability score), thus indicating that HL also influences work ability.

HL is regarded as a key component in disease management and an important target area for healthcare providers (Mackey *et al.*, 2016). At the provider–patient level, patient-centered communication, clear communication techniques, confirmation of understanding and reinforcement are intervention components considered important in improving HL and health behaviors (Sudore and Schillinger, 2009; Walters *et al.*, 2020). At the system–patient level, clear health education materials, and creating empowering and shame-free clinical environments, are some of the identified helpful intervention elements (Sudore and Schillinger, 2009). In work rehabilitation programs, research aiming to identify factors facilitating successful return to work and positive treatment outcomes emphasize several HL components: a clear explanation of health complaints, increased self-understanding regarding own identity, values and resources, a shared understanding of the health complaints with their healthcare provider and new learned behavior (Haugli *et al.*, 2011; Jakobsen and Lillefjell, 2014; Oosterhof *et al.*, 2014; Svanholm *et al.*, 2022).

### Study context and research needs

The outpatient clinic in the current study is part of the Norwegian government’s ‘Health and Work’ initiative. It is a brief work-oriented intervention provided through the secondary healthcare service, that is located in a nonhospital setting. It is comprised of a multidisciplinary approach involving follow-up by physicians, physiotherapists, psychologists, health educators and a job consultant from the Norwegian Labour and Welfare Administration (NAV). The brief intervention (BI) aims to contribute to better health and work ability among persons experiencing musculoskeletal and/or mental health complaints. The initial step of the BI is to perform a clinical examination of the patient to exclude serious pathology. When no serious pathology is discovered, the BI goes on to focus on normalizing the patients’ symptoms by providing insight, understanding and evidence for the benefit of staying active, thus facilitating adequate coping (Indahl *et al.*, 1995, 1998; Frederiksen *et al.*, 2017). Multiple randomized controlled trials have found the BI to be superior in facilitation of work participation when compared with more comprehensive interventions, both for persons with back pain (Karjalainen *et al.*, 2003; Jensen *et al.*, 2012; Reme *et al.*, 2016; Harris *et al.*, 2017) and persons with mental health complaints (Wormgoor *et al.*, 2020). However, studies providing in-depth information about participants experience with the BI is still scarce. Considering the prevalence and impact of musculoskeletal and common mental disorders, research should illuminate helpful intervention elements to tailor cost-effective and good quality interventions. Furthermore, research is needed to understand how experiences with healthcare services may influence the development, enhancement and application of health literacy skills (HLSs) (Squiers *et al.*, 2012).

### Study aim and theoretical framework

The current study aimed to explore what participants experienced as helpful elements in the BI, how they perceived them as helpful and the connection between BI and their subsequent coping. To sharpen the focus on intervention elements related to the acquisition and utilization of HLS, the HLS framework (Squiers *et al.*, 2012) was used to support the analysis. The HLS framework describes the pathway from the development and moderators of HLS to the application of HLS, and hypothesize relationships between HL and health outcomes, including both internal and external factors (Squiers *et al.*, 2012). The framework consists of four primary components, focusing on (i) factors that influence the development and use of HLSs, (ii) health-related stimuli, (iii) HLSs needed to comprehend the stimulus and perform the task and (iv) mediators between HL and health outcomes (Squiers *et al.*, 2012). The framework provides a useful lens to illuminate intervention elements of relevance for the development of HLS.

## MATERIALS AND METHODS

A focus group design was chosen as it enables communication and social interaction between participants sharing similar experiences (Morgan, 1997).

### Recruitment and sample

A purposive sample of patients, who had participated in the BI and had experienced the intervention as meaningful and helpful, was recruited.

The staff at the outpatient clinic identified patients who had experienced the BI as meaningful and helpful in their recovery process. These patients were then provided with oral and written information about the study and asked to consider participating in the research project. Patients who gave their written consent to participate were contacted by the project manager and invited to the focus group interviews. The sample consisted of five men and nine women, with age ranging from 43 to 58 years old (mean age = 51 years). Three of the participants worked full time, eight were partially back at work and three participants received sick leave benefits or were job seekers at the time of the interviews.

### Intervention

The BI at the ‘Health and work outpatient clinic’ is based on a noninjury model (Indahl *et al.*, 2009; Sorensen *et al.*, 2010), where complaints are not to be taken as a sign of injury caused by any wrongdoing or ‘inappropriate’ behavior, but rather as a normal part of life. The assumption that these common health complaints are inevitable features of human life is a core part of the BI (Eriksen *et al.*, 2004; Wilhelmsen *et al.*, 2007). Thus, the BI is better described as a ‘normalization approach’ rather than a curative approach.

The BI involved both individual and group sessions. Regardless of primary diagnosis and after exclusion of serious pathology, all patients were offered a group education (4 hr) focusing on normalization of their health complaints, debunking of common myths about these complaints and increasing participants’ HL on back pain, stress, common mental health complaints and their co-occurrence.

In addition, based on their situations and preferences, patients could choose to participate in brief psychotherapy, an examination by a physiotherapist or a medical doctor, a mastery-oriented group guidance (5 days) and/or individual guidance sessions. The intervention was flexible in that participants could choose to add courses or individual sessions with different professionals as long as they were registered as patients at the clinic. The focus on normalization of symptoms, redirecting patients’ concerns, reframing the current problems as facts of life, acceptance of the situation, guidance on how to handle the situation, restoring confidence in one’s own ability to produce a positive result and enhance awareness of their own resources and possibilities was consistent across the different treatment offers. The didactic approach was based on nondirective communication (Fisher *et al.*, 1997). Using this approach, the BI did not prescribe any change in lifestyle by telling patients what to do but aimed at establishing an understanding of common health complaints. The nondirective communication approach was used to demonstrate respect for participants’ autonomy and to reinforce confidence in their own capacity to discover and implement solutions on how to deal with health complaints and challenges. The aim of the BI was to facilitate a learning and change process contributing to increased coping and restoring or keeping the patients’ work ability. In the BI, coping was defined as a positive response outcome expectancy, as presented in the cognitive activation theory of stress (Ursin and Eriksen, 2004). Positive outcome expectancies refer to the belief that your actions will produce a desired result, meaning that the belief in the outcome of the strategies chosen is more important than the actual strategy itself. Positive response outcome expectancies are formed through learning and experiences (Ursin and Eriksen, 2004), and by strengthening participants’ expectancies of recovery from experienced health complaints the BI aimed to contribute to influence participants’ behavior and reduce the risk of long-term negative consequences.

### Data collection

Data were drawn from three focus groups, with a total of 14 participants (group 1 = six participants, group 2 = three participants and group 3 = five participants). The interviews took place at the outpatient clinic where the intervention had been carried out. Each interview lasted for 90 min and followed established focus group principles (Morgan, 1997). The interviews were recorded by using a recording device and later transcribed. The moderator (T.L.J.) invited the participants to share experiences and stories about how the intervention had made positive differences in their lives, including work participation, and how it helped them in coping with their health complaints or difficult life situations. A co-moderator (I.Ø.) took notes during the interviews, evaluated the atmosphere and interaction and asked follow-up questions when appropriate.

Sample size was guided by the assessment of information power (Malterud *et al.*, 2015). This included an assessment of the specificity of the research question and sample (the study had a specific research question and recruited a sample highly relevant to the study aim), the application of a relevant framework (the study was supported by The HLS Framework), the quality of the dialogue (all interviews had high dialogue quality and the participants shared plenty of experiences relevant to the study aim) and the analysis strategy (a thematic cross-case analysis presenting the participant’s experiences as they express them). After three interviews, we concluded that the information power of the sample was sufficient to conduct a responsible analysis. The interviews were audio recorded, encrypted and transcribed. Participants were assigned pseudonyms before transcription.

### Analysis

Systematic text condensation (STC), an explorative, thematic, cross-case method for qualitative data analysis, following a descriptive and stepwise approach, was used for the analysis (Malterud, 2012). STC has its origin from the descriptive phenomenological method of Giorgi, searching for the essence of the phenomenon while looking at objects from the perspective of how they are experienced (Giorgi, 1985, 2009). The analysis incorporated four steps. In *step 1*, the transcribed interviews were read by all authors to obtain a general impression of the data material and identify preliminary themes. In this process, the authors strove to consciously bracket out their preconceptions. All authors then met to share and discuss their general impression and agreed on three preliminary themes. In *step 2*, code groups were developed based on the preliminary themes from step 1 and units of meaning related to the code groups were identified. This was a comprehensive, nonlinear process where all authors worked together in identifying and categorizing meaning units in the transcripts, reflecting and negotiating on what each meaning unit was about and under which code it belonged. The number of code groups, and suitable naming of the groups, was also an issue in this step. In *step 3*, subgroups were established and the content in each of the coded groups was condensed. In *step 4*, the content of each code group was synthesized to present a reconceptualized description of each category. The two last steps were conducted through several rounds of discussions and comments between the authors. Agreement on four final core themes was reached (see results). The HLS framework (Squiers *et al.*, 2012) supported the analysis, however not as a template framework. The analysis was inductive and iterative, not theory-driven with prearranged coding.

The authors have a background in health promotion and health psychology, with a special research interest in HL, coping and the importance of work participation for health. Two of the authors (T.L.J. and I.Ø.) have clinical experience with the target group of the current BI and two of the authors (T.L.J. and T.H.T.) have previously participated in the implementation and evaluation of BI. We shared the preconception that the BI approach, by reducing uncertainty and faulty beliefs, would increase participants’ HLS, robustness and coping expectancies regarding their health complaints and work situation.

### Ethics

The Regional Committee for Ethics in Medical Research approved the study (REK sør-øst D, 283547). The study adhered to the Helsinki Declaration and was recommended by the Norwegian Social Science Data Services (NSD, ID 306154). Written informed consent was obtained from all participants.

## RESULTS

The analysis revealed multiple aspects of participants’ perceptions of helpful elements in the BI, how they perceived them as helpful and the link between the BI and their subsequent coping. The participants emphasized the importance of having the information and guidance presented in a comprehensible way, using metaphors, recognizable narratives, practical examples and exercises. Normalizing and demedicalizing their situation defused frightening thoughts, which together with a safe and accommodating clinical environment, facilitated change processes. The BI initiated processes contributing to acceptance, resilience and empowerment, aiding both work–life balance and return-to-work processes. The findings are expanded upon in the text below. The connection to HLS enhancement is elaborated in the discussion.

### Information and guidance delivered in a comprehensible way contributed to awareness, understanding and behavioral change

The participants claimed that the intervention increased awareness and understanding of the health complaints and how to live with them. They saw this as a result of the thorough explanation provided in the BI about relevant anatomy, physiology and consequences of their health complaints. One participant struggling with tiredness told how the information about stress (explaining load, overload, sustained activation and the stress response), had been an eye-opener to her. Before participating in the BI, she worried about activities that made her tired because she thought this would increase her recovery time. When she understood that abstaining from activities she enjoyed doing was not a good way to recover from tiredness, she gradually started to challenge herself and experienced that her energy returned. She believed that her breakdown might have been prevented if she had received this information earlier. Others told similar stories, emphasizing that debunking the common metaphor of the body as a battery recharging through rest and sleep was an ‘aha’ experience for several of the participants. Furthermore, the participants experienced their new knowledge as empowering, as expressed by one of the participants:

*…it was a part of my treatment, that my shoulders just dropped when they talked about these things. Because I got an explanation of what I was struggling with.*

The use of metaphors was perceived as important for making the information coherent and comprehensible, and easier to remember. One participant explained how one of the metaphors had made a lasting impression and helped her to reflect on her situation. The metaphor pictured a river as the course of life, where sometimes you float with the river and sometimes you need a break on the riverbank. She explained how she had placed herself in this picture, sitting on the riverbank, worried that she would stay there forever. At that point she did not know how or when to get back in, or what it would look like if she did. But she realized that jumping back into the river without changing anything, probably would produce the same result all over again. Her job was to find out if she should adjust to the current river flow by finding new ways to swim or navigate to a place where the river was calmer. She explained that the metaphor provided hope:

*I understood that this was for a limited period of time. I was going to get into the river again, but maybe in a different way. … This was by no means final.*

In addition to the metaphors, the participants gave practical examples of how the exercises helped them forward by changing how they viewed their own thoughts and actions. One exercise asked them to describe what a typical day in their life looked like, then to compare this with a description of an optimal day. Several participants said that comparing the actual situation to a more desirable situation made them more aware of how much time they used on things like scrolling on their phones or watching TV, and where they could implement changes. Collaborating with other participants when performing these exercises provided a wider range of ways to understand and solve a problem. The introduction to practical ways of handling thoughts, e.g. implementing a specific time for rumination, was perceived as very useful. They were surprised about how awareness and practice allowed them to influence their thoughts. A quote from one of the participants illustrates the impact of the intervention on her subsequent coping:

*This was just what I needed. In a way, it was like getting antibiotics for an infection. Now I have some tools and know what to do.*

The intervention contributed to behavioral changes such as coming to grips with challenging job situations, seeking new jobs and implementing changes in their work schedules. Another useful change was the implementation of more positive words and language. The participants gave examples such as changing from using the words ‘I should’ or ‘I have to’ to the words ‘I choose to’ or ‘I want to’. In this way, they took ownership of their change processes and acknowledged that they had done most of the work themselves. They had received help and support but emphasized the importance of their own engagement in changing their mindset and their behavior. One of the participants expressed that this was his most valuable lesson and summarized his experience as follows:

*I believe that this information should be the syllabus for everyone. … I realized that it is not about pulling yourself together. I had to do something else. Then things kind of fell into place.*

### Normalization, demedicalization, recognition and psychological safety were change facilitators

There was agreement among the participants that a demedicalization of their experienced health complaints through information and normalization had made their situation less frightening. Information about how your mind may trick you in challenging periods of life was both recognizable and useful. One participant explained how he had been frightened by his own negative thoughts, leading to the assumption that his condition was very serious. Normalizing these thoughts helped to defuse his situation. The participants furthermore emphasized the importance of recognizing their own situation in the information presented in the BI. In this way, the intervention made them confident that their complaints were real and not imagined. The information provided in the first course was perceived as so relevant to their own experiences that they almost felt the lecturers were talking about them. One helpful example was of a sick-listed man who was ashamed of going grocery shopping during normal working hours, because then other people would know he was sick-listed. One participant said this was exactly the way he had felt. He had driven to stores far away from his home to avoid running into people he knew. Because of the recognition in the information and examples provided in the course he felt he was at the right place:

*I know that there are many people who struggle with depression and anxiety and these things, but I recognized myself so much in what I heard on the first course … It gave me an aha experience. Then I had the courage to go all in on the rest of the scheme.*

New insight was achieved about the normality of experiencing health complaints and challenging periods in life, both through information in the course and meeting other participants in similar situations. A participant who mostly worked with younger colleagues who never complained about their health felt less alone when she saw how others struggled, and this relieved her bad conscience. Getting rid of the perception that they were weak and not able to handle pressures and strain and accepting that they struggled, was an important part of the process. Acceptance was repeatedly mentioned, both regarding experienced health complaints and being sick-listed from work. Several of the participants mentioned that it would have been easier to accept their situation if their health complaints had been visible, something physical, like a broken leg. Going to the store during regular working hours with a broken leg would not have been a problem but with invisible health complaints it was much harder. It was perceived as easier to talk about physical than mental complaints, e.g. starting by seeing a physiotherapist about back pain could be a useful first step, even when knowing that the situation comprised more than back pain. The threshold for seeing a psychologist was high among several participants. Having mental health complaints and not coping was initially hard to accept but the normalization they experienced through participating in the intervention helped them both to accept and cope. One of the participants said:

*You’re not unique in feeling this way. But I never thought it would happen to me. So, I was kind of relieved when I left … I felt, okay, this is actually quite normal.*

The participants felt safe and listened to at the outpatient clinic and in their encounters with the health professionals. They perceived the health professionals as confident, inclusive and highly competent in their field. One participant had for a long time denied that he had mental health complaints due to feelings of defeat and shame. When he finally admitted that he needed help and decided to share his problems, feeling safe in the treatment setting was very important to him. Other participants emphasized the importance of being seen for who they were and not for their illness. They were met with openness, care and respect, and appreciated not being medicalized. The safe, but vulnerable, group setting in the course had been instrumental for one participant in her process of navigating back into society. Another participant shared an experience from her meeting with the psychologist, that had made her feel safe and more relaxed:

*Early on, I was told that you shouldn’t come here thinking you’re going to be a clever patient. It was actually very helpful that the psychologist said this. That you are not here to perform. That was very important for me to hear.*

### The initiated processes contributed to acceptance, resilience and work–life balance

The course helped the participants to accept and interpret their health and work situation in a better way. The intervention gave them time and space to find new solutions to their composite problems and to rest and recuperate. A common challenge among the participants was setting limits for themselves. The participants got help to rebuild their boundaries not only by speaking up and by tempering their expectations of themselves but also by getting confirmation that setting limits for oneself is all right, without feeling less valuable. This perceived legitimacy and balancing of different aspects of life were expressed as an important road to recovery and return to work. Several participants felt shameful because they were not able to work and put a lot of pressure on themselves to return to work. Hence, they had thought that the staff at the outpatient clinic would start pushing and making demands for a quick return to work but they felt no such pressure. They experienced the outpatient clinic as a place where they could get respite and gather strength before jumping back to navigate all the demands again. It helped them to hear that stressing about their reduced function could be counterproductive, and that they were allowed to focus on functioning as human beings 24 hr a day, not only at work. One participant explained how the balance of getting time to recover away from work, but at the same time getting inspiration to do something useful when recovering, was important to him:

*The therapist I went to before was a bit sceptical about my stay at the Health and Work clinic because he thought I very quickly would enter a race where the goal was to go straight back to work. What you need is time, he said. But I had loads of time – the problem was that I was not able to use that time to do anything useful.*

### A simultaneous focus on work and health aided the process of return to work

The simultaneous focus on both work and health was very much welcomed by the participants. Having a job was important to them; work provided both well-being and meaning in life. They were dedicated to doing a good job; hence, their work ability was an important and meaningful target area. The intervention provided insight into how closely intertwined work and health are. One participant had expected to be told when she was considered 100% healthy and thus ready for returning to work, but during the intervention she realized that the last part of the rehabilitation takes place by participating in working life again. Another participant had realized that dealing with the demands and stressors in working life was essential for her recovery and coping:

*The physician who sick-listed me meant that my work was the cause of most of my stress. But here at the clinic, they had a different approach … Although I don’t like stress, I also appreciate a bit of stress … So, the lesson here, about how to deal with stress was important to me. Because we are in this world, and there are always stressors in various ways.*

Many of the participants appreciated the help they got from the NAV-consultant engaged at the clinic. Some described it as lifesaving first aid. This was help they had not received through the ordinary NAV system, mainly because of the organization’s extended use of digital platforms which they had trouble navigating. They experienced it as challenging to reach NAV by phone and described how the practical part of being on sick leave could be experienced as very stressful. They were worried about doing something wrong and losing their benefits. The letters they received from NAV were hard to understand and the wording came across as alienating, threatening and unfriendly. Thus, it was a great relief to be able to speak with the NAV-consultant at the outpatient clinic. She made the sick leave process and the various requirements from NAV simple and harmless, in addition to contributing with arrangement of dialogue meeting with employers when needed. One of the participants described her previous feelings about NAV, and her positive experience with having a NAV-consultant available at the outpatient clinic, in this way:

*Being on sick leave is actually a job in itself. Because for me, every time there was a message from NAV or something like that, I felt a knot in my stomach …then my whole day was ruined. After talking with her (the NAV-consultant at the Health and Work clinic), it became disarmed.*

## DISCUSSION

The results indicate that the BI may contribute to improving patients’ musculoskeletal and mental HL. The participants highlighted the importance of normalizing and demedicalizing subjective health complaints through health education, achieved through using a comprehensible language entailing metaphors and recognizable narratives. This contributed to knowledge and a more fundamental understanding of experienced health complaints, thereby diffusing frightening thoughts and worries. These factors, together with perceiving the clinical environment as safe and receiving person-centered support when needed, were highlighted as important in facilitating change processes. The simultaneous focus on work and health, and the importance of staying active despite health complaints, provided hope and positive expectancies regarding future function and work participation. Resilience and empowerment appeared as capabilities strengthened through participation in the BI.

There is agreement that the way people view health and illness influence their behavior (Hughner and Kleine, 2004; Petrie and Weinman, 2006, 2012). Hence, the reframing of people’s health and illness perceptions have the potential to alter emotional responses, coping behavior, clinical outcome and work participation (Petrie *et al.*, 2002; Petrie and Weinman, 2006; Foster *et al.*, 2008; Løvvik, Øverland, *et al.*, 2014; Løvvik, Shaw, *et al.*, 2014). In this study, there were multiple examples where participants had changed their understanding of health complaints and how the body works. One example presented as an ‘aha’ experience by several of the participants, and which changed their view of recovery from fatigue and tiredness, was debunking the common myth describing the body as a battery recharging only through rest and sleep. Furthermore, the demedicalization of health complaints through information and normalization appeared as one of the most helpful elements in the intervention, giving the impression that participants previous views primarily were based on a biomedical understanding of health and illness. A medicalization of normal behavior and subjective health complaints may disempower people and decontextualize personal experiences (Mulder, 2008). Educational interventions informing people about the normal presence of health complaints and bodily distress in healthy people may thus be highly relevant to facilitate recovery and coping (Barsky and Borus, 1995; Mulder, 2008). In this study, the normalization approach in the BI, in addition to the resource orientation acknowledging patients’ ability for self-management and decision-making, emerged as elements which contributed to a reduction of self-stigma and a strengthening of participants’ resilience and empowerment. These are conceptually intertwined concepts, but there is robust evidence for the negative relationship self-stigma has on empowerment and recovery (Livingston and Boyd, 2010; Oexle *et al.*, 2018). Reducing self-stigma is thus an important target area in the management of subjective health complaints. The strengthening of patients’ empowerment is furthermore highlighted as an essential activity in the integrative model of patient-centeredness, in addition to information, involvement in care and support (Scholl *et al.*, 2014), all of which have a central role in the participants’ descriptions of helpful elements in the BI.

### Intervention elements related to the acquisition and utilization of health literacy skills

Comprehension of the delivered information is a primary indicator of HL and hence a prerequisite to the development of HLS (Squiers *et al.*, 2012). To facilitate comprehension, the orientation of the message and the language used are important elements (Squiers *et al.*, 2012). These elements were clearly expressed by the participants: it was not just the information provided that mattered, but also how the information was delivered. They appreciated the demedicalization and resource orientation, and the use of a plain and comprehensible language peppered with metaphors and examples that made the information easier to remember and relate to. Although comprehension and message characteristics are essential to determine what someone will learn, it is not sufficient to directly affect health behavior and health outcomes (Squiers *et al.*, 2012). Mediating factors, such as trust in the information and its source, perceived relevance of the message, perceived effectiveness and social support may affect whether participants accept and adopt what they have learned (Squiers *et al.*, 2012). In this study, the participants described how they had acted on the information provided and implemented several changes. When looking at the study results using the HLS conceptual framework (Squiers *et al.*, 2012), important mediators for the development of HLS are present. Identifying mediating factors in the participants’ descriptions may provide useful knowledge for future tailoring of BI.

One important mediating factor raised by the participants was how the credibility and communication skills of the healthcare providers played a major role in influencing their ability to process and interpret the provided health information. This highlights the importance of trusting the health professionals and perceiving them as confident and competent in their work and communication. This finding is in accordance with results from a previous study investigating back pain patients’ experiences with the BI, where comprehensible information and perceiving the healthcare providers as experts emerged as important factors in facilitating patient coping (Ree *et al.* 2014). In addition to trusting the message of healthcare providers, relevance of the message was very important to the participants. This was expressed as recognition and highlighted as a factor contributing to a feeling of being in the right place and to the belief that the intervention would be helpful for their recovery process. When this recognition arose early in the intervention, it facilitated the decision to complete the rest of the intervention and accordingly appeared as an important factor to focus on in the start of BIs.

Social support was expressed both in relation to meeting other participants struggling with similar problems and in the contact with healthcare providers. Several of the participants had benefited greatly from the group settings because they met like-minded people who openly shared their experiences and saw that they were not alone in having problems. They appreciated how the individual and group approaches complemented each other, each providing benefits the other would not. The healthcare providers were perceived as inclusive and person-centered, treating them as equal partners in the recovery process, but still providing adequate support and expertise knowledge. They described this balance as an empowerment and coping facilitator, giving them the realization that they themselves had the answers to many of their problems and actually had done most of the work themselves.

HL includes the ability to navigate complex social and health systems (Squiers *et al.*, 2012). In this study, the participants especially talked about how the NAV system was a negative additional load in their sick leave period, a load that for some of the participants was experienced as a hinder in the recovery process. The establishment of the cooperation between the NAV, a key nonclinical stakeholder in the return-to-work process, and the outpatient clinic providing the BI was much appreciated by the participants. Participants who communicated with the NAV-consultant at the outpatient clinic had similar experiences as in their meetings with the healthcare professionals: normalization, trustworthy information and diffusion of frightening thoughts. The inclusion of a NAV consultant in the outpatient clinic was fairly new and had not been available to all the participants in this study. Several of these participants would have used it if it had been available. Based on the findings in this study, the cooperation between, and co-location of, employees representing both the health and welfare systems may be an important facilitator for return to work.

## STRENGTHS AND LIMITATIONS

The participants openly shared their narratives and experiences with the BI, giving the impression of trustworthy and authentic stories of how and why the intervention was perceived as useful. The focus in the interviews was to identify helpful elements, but the participants also presented challenges and possible improvements, indicating that they felt safe to report undesirable experiences as well. The interview material was rich and diverse, with good information power, and we consider our findings to be a valuable contribution to the research literature on patients’ experiences with the BI targeting musculoskeletal and mental health complaints and factors facilitating the recovery process. In a previous focus group study, Ree *et al.* (2014) raised the hypothesis that the impact of important aspects of BI on back pain patients’ coping might be transferable to patient groups suffering from other subjective health complaints. The current study supports this hypothesis, revealing that patients struggling with mental health complaints and tiredness recalled similar stories and presented a lot of the same important aspects. As this study consisted of a strategically selected sample of patients having positive experiences with the BI, additional research will be required to shed light on the experiences of less successful participants and what they perceived as challenging or potentially provoking intervention elements. Future research projects could also strive to recruit participants from different BI clinics to increase sample diversity and population validity.

The results from the study are of value for the tailoring of future BI and give insight into which elements it may be useful to further cultivate. The study’s focus on identifying intervention elements to promote the development, enhancement and application of HLS, contributes to filling this knowledge gap. The study highlights important elements mediating HL enhancement, which may be useful to guide the practice of different stakeholder working with return-to-work interventions.
